# Do calves hide after birth? Postpartum behavior of dairy calves and their dams housed in individual calving pens

**DOI:** 10.3168/jdsc.2022-0364

**Published:** 2023-10-03

**Authors:** Margit Bak Jensen, Guilherme A. Franchi, Maike Schumacher, Kathryn Proudfoot

**Affiliations:** 1 Department of Animal and Veterinary Sciences, Aarhus University, DK-8830 Tjele, Denmark; 2 Atlantic Veterinary College, University of Prince Edward Island, Charlottetown C1A4P3, Canada

## Abstract

Under near-natural conditions, domesticated dairy calves hide the first days after birth before cow and calf join the herd. In commercial dairy production, an opportunity to seclude from the herd after parturition is rarely given. This study aimed to investigate the effect of providing a covered area in an individual calving pen on maternal and neonatal calf behavior. Forty-six cow-calf pairs were housed in either an individual uncovered calving pen with 4 open sides or an individual partially covered calving pen with 3 covered sides, providing a secluded area for the cow and calf. Calf position in the pen, maternal behavior, and proximity between the cow and calf were recorded for the first 72 h after birth using instantaneous sampling at 5-min intervals. Data were analyzed using linear mixed effect models. The duration of maternal sniffing and licking, the duration of time the cow spent standing with her head over the calf, and the time spent in close proximity to the calf were higher during the first 24 h after birth compared with later days, reflecting intense maternal behavior during this early period. Calves did not show a preference for staying in the covered side of the pen. Calves in covered pens received more maternal sniffing and licking, indicating that the provision of cover postpartum facilitated maternal behavior and the formation of the maternal-filial bond the first few days after birth.

## Highlights

• Calves did not prefer to stay in the covered side of the pen after birth.
• Calves in covered pens received more maternal sniffing and licking, suggesting that the provision of cover facilitates maternal bond formation.
• The duration of maternal behavior, including maternal protective behavior, was higher during the first 24 hours after birth compared with later days.

## Graphical Abstract

**Figure:**
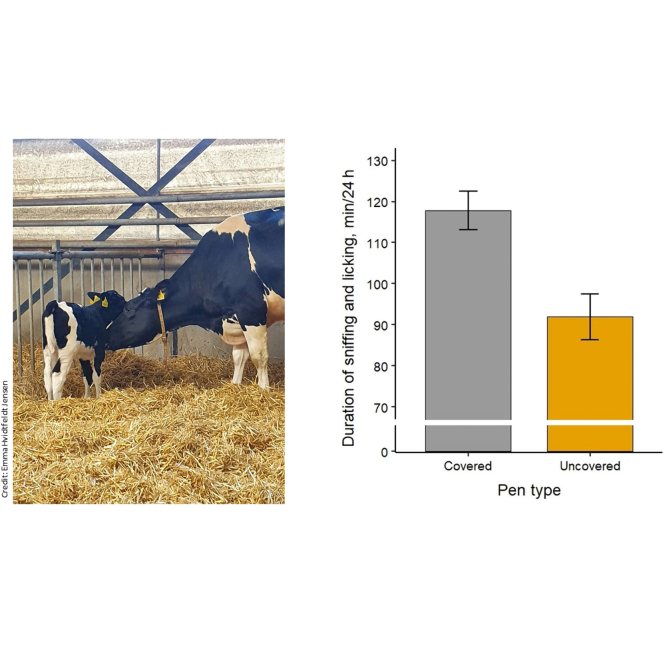
**Summary:** This study investigated the effect of providing a secluded area in an individual calving pen on maternal and neonatal calf behavior during the first 3 days after birth. Although calves did not spend most of their time in the secluded area of the pen, calves in covered pens received more maternal sniffing and licking compared with those that did not have a secluded area. This finding indicates that providing cover after birth facilitates maternal behavior and the formation of the cow-calf bond. Maternal behavior and protective behavior were more intense during the first 24 hours after birth than later days. These results provide evidence that dairy cow-calf pairs may benefit from the provision of a secluded area where they can bond during the first days after the birth.

On most dairy farms, it is common to separate the cow from her calf immediately after birth, which prevents the pair from performing natural maternal and neonatal behavior. This practice is not in line with consumers' expectations for animal welfare (Ventura et al., 2016; Hötzel et al., 2017), and there is therefore an increasing interest in dam-rearing calves (Johnsen et al., 2016). However, one challenge of this practice is to ensure the establishment of a strong maternal-fillial bond. Providing the cow and her calf with an environment that offers an opportunity to seclude from the herd may facilitate maternal-filial bonding after birth.

Under near-natural conditions, cows typically seek seclusion from the herd to give birth and to bond with the neonate. During the first days after birth, the calf hides in vegetation or tall grass while the cow forages nearby (Vitale et al., 1986; Langbein and Raasch, 2000). However, this seclusion from the herd is not always seen in near-natural environments; although most correctly classified as “hiders,” cows only seclude from the herd when the environment contains suitable hiding places for parturition and for the calf after birth (see Rørvang et al., 2018b, for review). In recent years, studies have investigated how the environment may facilitate seclusion at calving (e.g., Proudfoot et al., 2014; Rørvang et al., 2018a), but little is known about how to design environments that facilitate cow-calf bonding.

Another crucial aspect for the survival of the neonate is maternal protection. If a dam is exposed to a potentially dangerous situation during lactation, she remains close to her calf, and if perceived necessary, she will perform aggressive defenses to protect her young (Flörcke et al., 2012). However, maternal protectiveness impedes the handling of cow and calf and poses a risk of injuries to stockpeople and the calf itself (Örnehult et al., 1989; Edwards et al., 1995). In a near-natural environment, cows approach their calves mostly for nursing or in situations of imminent danger (Vitale et al., 1986), and Kiley-Worthington and de la Plain (1983) reported that when the cow and calf spent time together, the cow would often stand over the lying calf. It is not known whether allowing the calf to hide during the first days after birth may reduce cow protectiveness.

Recent studies investigated the effect of providing hides on cow behavior before and during parturition and they found that the hides were also used by the calves after calving. For instance, Jensen and Rørvang (2018), found that only 6 out of 58 cows calved in a provided secluded area, while 19 out of 36 cow-calf pairs entered the secluded area within 3 h after birth. In accordance with this, Zobel et al. (2020) found that in addition to 20% of the cows moving into hides before calving, a further 40% to 60% of the cow-calf pairs moved into hiding places soon after birth. These results suggest that providing hiding places to calves after birth potentially provides them with the opportunity to perform their natural hiding behavior.

This study aimed to investigate the effect of providing a partially covered area in an individual calving pen on maternal and neonatal behavior of dairy cattle. We hypothesized that calves housed in a partially covered pen would spend more time on the covered side of the pen compared with the open side. Furthermore, we hypothesized that calves housed in a partially covered pen would spend more time lying down due to feeling more calm and receive more maternal care than calves housed in the uncovered pen due to a potentially less-disturbed bonding process. Additionally, we hypothesized that cows housed in a partially covered pen would allow more distance to their calves and show less protective behavior due to a potential experience of safety.

The experiment was conducted at the Aarhus University cattle research facility (Viborg, Denmark) between September 2011 and February 2012 and in accordance with a protocol approved by the Danish Animal Experiments Inspectorate (journal no. 2010/561–1780). Previous publications based on the same experiment reported the effects of time of movement to individual calving pens (Proudfoot et al., 2013), the effect of pen type on prepartum behavior (Proudfoot et al., 2014), and the effect of disease on maternal behavior (Perier et al., 2019).

Animals and housing are described in detail in Proudfoot et al. (2014). In short, 79 multiparous Danish Holstein dairy cows were enrolled in the experiment in 6 blocks. At least 10 d before the expected calving of the first cow in a block, cows were moved into 1 of 2 group pens (each 9 × 15 m) with deep straw bedding. Each block included at least 1 nonexperimental cow or heifer, which had expected calving dates later than the experimental cows to ensure that at least 1 cow was present in the group pen.

Within block, the cows were moved into 1 of 10 individual calving pens either 3 d before expected calving, or when they showed sign of imminent calving (e.g., relaxed pelvic ligaments, signs of bloody mucus), and were alternately assigned to either an uncovered or a partially covered individual calving pen. See Proudfoot et al. (2014) for a diagram of the pens. Each pen was 3 × 4.5 m large and was surrounded by 1.3-m-high tubular metal bars on 3 sides and a feed bunk on the fourth side, which was facing the outer wall. In half of the pens (partially covered), a 1.8-m-high plywood barrier fully covered 2 pen sides, and the side facing the group pen was half covered to create a secluded area (referred to as the “covered side”) and a 1.5-m-wide window (referred to as the “open side”) that allowed visual contact to the group pen. Each partially covered pen was matched with one uncovered pen, which acted as a control to determine the “covered side” in the uncovered pen. The uncovered pens allowed visual and head-to-head contact with cows in the group pen and cows and calves in the adjacent individual pens. After calving, the cow and calf remained together for at least 3 d in the individual calving pen.

Twice daily at 1000 and 1700 h, cows were fed ad libitum with a TMR with a forage-to-concentrate ratio of 79:21 (%, DM basis) before calving and 60:40 (%, DM basis) after calving. All cows had ad libitum access to water. The cows were milked twice daily at 0600 and 1800 h by a manual milking machine in the calving pen. The pens were provided with fresh straw bedding once daily.

All calves received colostrum within 6 h after birth. From the calves included in the study (n = 46, see inclusion and exclusion criteria below), 29 received colostrum with a bottle, 14 calves suckled directly from the cow, while this information was not recorded for 3 calves. Tube feeding was never performed. For 19 calves, suckling was assisted at least twice. The number of included calves was supported by power calculations to detect significant differences at α = 5% between the proportion of calves seeking secluded areas with a narrow (59%) and a wide barrier (30%) with a power of at least 80% (proportions were calculated from Table 2 in Jensen and Rørvang, 2018) using the R package *pwr* v.1.3–0 (Champely, 2020).

This study included only healthy calves from cows that were healthy and experienced a normal, unassisted birth in an individual calving pen (see Proudfoot et al., 2014, for selection criteria). Of the 79 enrolled cows, a total of 12 cows were excluded because of illness; cows were considered ill if they had at least 2 consecutive body temperatures >39.0°C and showed signs of metritis (n = 3; a score ≥2, all diagnosed on d 3 after calving), mastitis (n = 5; 3 diagnosed on d 2 after calving and 2 diagnosed on d 3 after calving), pneumonia (n = 2; diagnosed by a veterinarian on d 2 after calving), or a combination of mastitis and metritis (n = 2; diagnosed on d 2 and 3 after calving, respectively). Furthermore, cows were excluded if they calved in the group pen (n = 10), experienced a difficult calving or had twins (n = 7), were disturbed by human adding straw during labor (n = 1), or had milk fever on the day of calving (n = 2). One calf was excluded due to signs of illness. Thus, a total of 46 cow-calf pairs were included in the study; 19 were housed in an uncovered pen and 27 were housed in a partially covered pen.

One experienced observer recorded all data from video. The behavior of each calf was observed from the moment of calving until 72 h after calving using 5-min instantaneous sampling (Bateson and Martin, 2021). At each sampling point, the location of the calf in the pen, the proximity of the calf and the cow, the posture of the calf (standing or lying), and behaviors displayed or received by the calf were recorded (see Table 1).

**Table 1:** Ethogram of calf posture and behavior (active and passive) organized in 4 levels: calf position, proximity of cow and calf, calf posture, and calf behavior^1^

Behavior	Level	Definition
Open side	Position	The calf was in the open side of the pen when the majority of its body was positioned in this area. If the calf was positioned in the center of the pen, it was considered to be on that side where its head was positioned.
Covered side	Position	The calf was in the covered side of the pen when the majority of its body was positioned in this area. If the calf was positioned in the center of the pen, it was considered to be on that side where its head was positioned.
Near the cow	Proximity to the cow	Cow-calf distance ≤1 m.^2^
Away from the cow	Proximity to the cow	Cow-calf distance >1 m.^2^
Lying	Posture	The calf is lying on sternum or on side with head resting or raised.
Upright	Posture	The calf's body is supported by 4 legs; the calf is standing or walking.
Suckling dam	Behavior	The head of the calf is under the cow's belly in the udder area.^3^
Being sniffed or licked on the head or body by the cow	Behavior	The muzzle of the cow is in contact with, or in close proximity of, the calf's head or body.^4^
Being sniffed or licked and suckling at the same time	Behavior	The calf suckled and was sniffed and licked by the cow at the same time.^4^
Cow's head over calf	Behavior	The calf's body is covered by the cow's head. The cow is lying or standing, while holding her head over the calf.^4^
Being shielded by cow	Behavior	All sides of the calf are shielded by either the cow or the walls. Calf and cow are either lying or standing.^4^
Other activity	Behavior	All activities that were not mentioned above.
No activity	Behavior	No apparent activity.
Human disturbance		Human inside the pen.

1 Within level, the postures and behaviors were mutually exclusive.

2 The distance was determined based on the closest distance between cow and calf were chosen, regardless of the body part.

3 Active behavior: performed by the calf.

4 Passive behavior: performed by the cow and directed to the calf.

Based on the instantaneous recordings, the durations (min/24 h) were estimated for the 4 levels: position, posture, proximity, and behavior using SAS 9.4 (SAS Institute Inc.). Due to short periods of missing video recording, observation time was reduced for 20 calves (42 ± 24 min/calf over 3 d). In addition, the duration of human disturbance was excluded from the behavioral observations (33 ± 19 min/24 h). Thus, the duration of daily recordings per calf was, on average, 1,387, 1,405, and 1,411 min on d 1, 2, and 3, respectively.

The response variables were grouped according to the 3 research aims described below.

Aim 1: *whether calves show a preference for the covered side*. This response variable was the duration of more time spent in the covered side (or corresponding matched side in uncovered pens). To account for the varaiblilty in reduced observation time among calves (see above), this response variable was calculated as the time calves spent on the covered side subtracted by half of the total observation time (i.e., the sum of time on the covered and open side).

Aim 2: *whether the pen type affects the duration of behaviors, including whether calves housed in partially covered pens received more maternal behavior*. The response variables were calf suckling, cow sniffing and licking, protective behavior 1 (cow standing head over calf), protective behavior 2 (cow shielding calf), and calf lying time.

Aim 3: *whether the pen type affects the time calf spent close to the dam.* The response variable was the daily duration cow and calf spent at a distance of less than 1 m from each other (time spent close).

Statistical analyses were performed in R v.4.1.1 (R Core Team, 2021) using the calf as the experimental unit. All variables were initially analyzed with linear mixed-effects model with a Gaussian distribution (library *glmmTMB* v.1.1.2; Brooks et al., 2017), including pen type, day, and pen type × day interaction as fixed effects. Calf and block were included as random effects. Additionally, a continuous-time autoregressive covariance structure was included to account for repeated measures of each calf over days. Calf lying time was square-root-transformed to fulfill the assumptions of normality and homoscedasticity. Because of the percentage of zero observations (17%), “protective behavior 1” was square-root-transformed to fulfill the assumptions of normality and homoscedasticity. Due to the high percentage of zero observations (80%), “protective behavior 2” was converted into a binary variable (received; not received) and analyzed with a mixed-effects logistic regression model (library *glmmTMB* v.1.1.2). Model assumptions of normality and homoscedasticity were confirmed through graphical inspection of the residuals. Tukey-adjusted post hoc analyses were performed for each statistically significant effect (library *emmeans* v.1.6.3; Lenth, 2021) and estimates are presented as least squares means and standard error, unless otherwise stated.

The results showed no interaction between pen type and day for any of the variables. Below we present the main effects of treatment and day for the variables relating to each of the 3 aims.

Aim 1: The duration of time that calves spent in the covered side, or corresponding matched side in uncovered pens, did not differ between partially covered pens and uncovered pens (89 ± 33.7 vs. 10 ± 40.2 min/24 h, respectively; χ^2^_1_ = 2.27; *P* = 0.132). Similarly, no significant difference among d 1, 2, and 3 on time spent in the covered side, or corresponding matched side in uncovered pens, was detected (Table 2).

**Table 2:** Behavior of dairy calves housed in an individual calving pen with either partially covered or uncovered sides on d 1, 2, and 3 after birth^1^

Data from video recordings	d 1	d 2	d 3	χ^2^_2_	*P*-value
More time spent in the covered side (aim 1)^2^	65 ± 45.4	48 ± 45.4	34 ± 45.4	0.29	0.865
Time spent suckling (aim 2)^2^	51 ± 3.9	44 ± 3.9	52 ± 3.9	3.56	0.168
Time receiving sniffing and licking (aim 2)^2^	162 ± 6.4	73 ± 6.4	80 ± 6.4	129.53	<0.001
Time receiving protective behavior 1 (aim 2)^2^, ^3^	73 ± 10.7	39 ± 7.8	21 ± 5.8	30.74	<0.001
Time receiving protective behavior 2 (aim 2)^4^	0.2 ± 0.12	0.2 ± 0.11	0.2 ± 0.10	0.36	0.835
Calf lying time (aim 2)^2^, ^3^	1,122 ± 9.3	1,195 ± 9.6	1,149 ± 9.4	79.87	<0.001
Time spent close to dam (aim 3)^2^	1,196 ± 23.9	1,049 ± 23.9	959 ± 23.9	88.1	<0.001

1 Estimates for the main effect of day are given.

2 Results are presented as LSM ± SE (min/24 h).

3 LSM ± SE back-transformed from the square-root scale are displayed.

4 Results are presented as probability ± SE.

Aim 2: Suckling duration did not differ between calves kept in partially covered pens and uncovered pens (46 ± 3.8 vs. 52 ± 4.5 min/24 h, respectively; χ^2^_1_ = 0.93; *P* = 0.336). Similarly, no significant difference among d 1, 2, and 3 on suckling duration was detected (Table 2).

Calves housed in partially covered pens received more maternal sniffing and licking than calves in uncovered pens (χ^2^_1_ = 12.15; *P* < 0.001; Figure 1). Additionally, calves received more sniffing and licking on d 1 than on d 2 and 3, regardless of pen type (Table 2).

**Figure 1:**
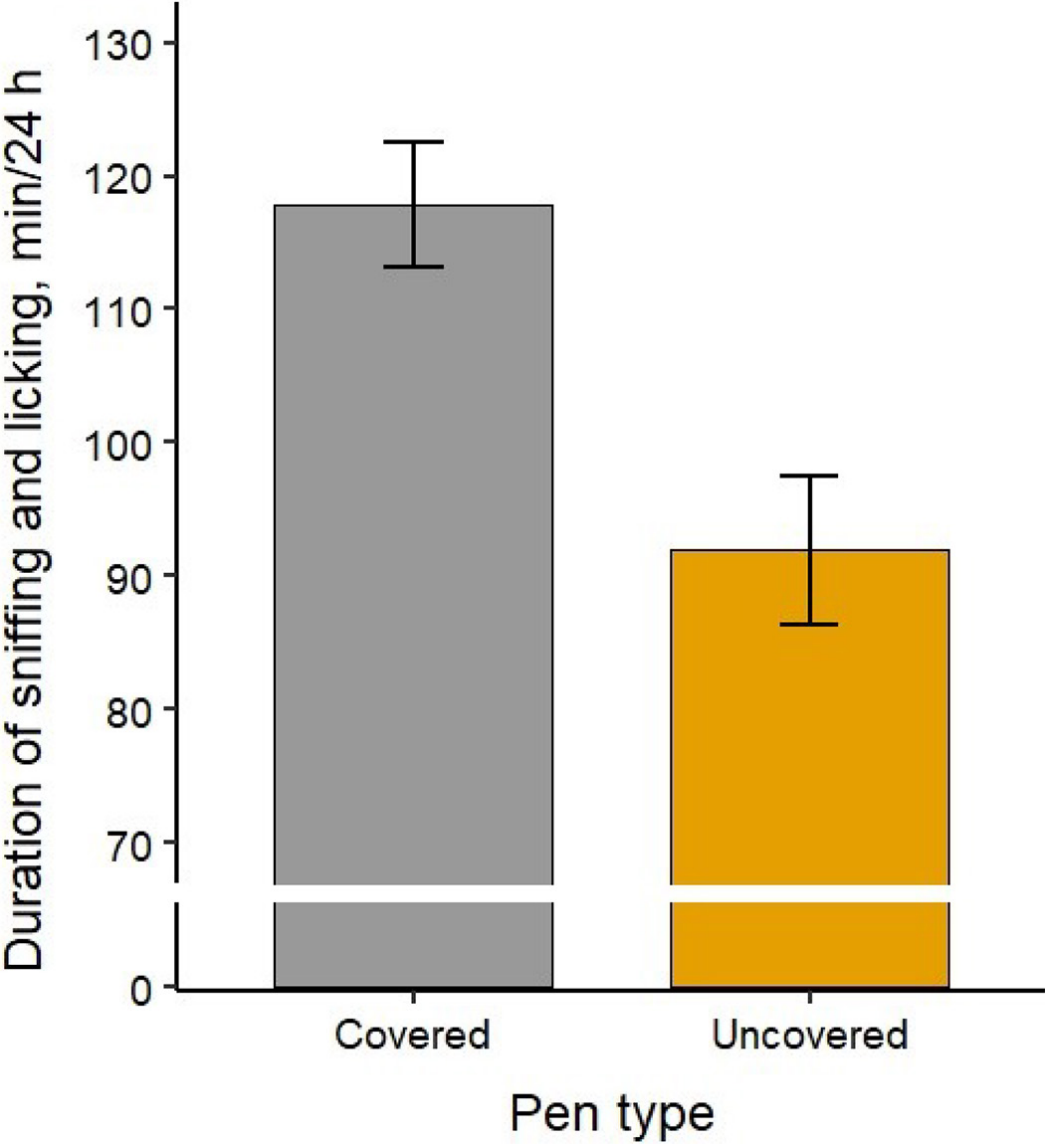
Duration of sniffing and licking received by calves in each pen type. Bars represent LSM, and error bars indicate SE. Different bar colors indicate a significant difference at *P* < 0.05.

Calves received more protective behavior 1 (cow standing head over calf) on d 1, followed by d 2 and 3; Table 2), regardless of pen type. Protective behavior 1 duration did not differ between partially covered pens and uncovered pens (38 ± 7.8 vs. 45 ± 10.2 min/24 h, respectively; χ^2^_1_ = 0.23; *P* = 0.627).

The odds of calves receiving protective behavior 2 did not differ between partially covered pens and uncovered pens (odds ratio ± SE: 2 ± 0.9; χ^2^_1_ = 0.37; *P* = 0.542). Similarly, no significant effect of day was detected (Table 2).

An effect of day on calf lying time was detected (Table 2). Calf lying time did not differ between partially covered pens and uncovered pens (1,165 ± 10.2 vs. 1,145 ± 12.0 min/24 h, respectively; χ^2^_1_ = 1.47; *P* = 0.226).

Aim 3: Cows and calves were in close proximity for longer on d 1, followed by d 2 and 3 (Table 2), regardless of pen type. The time calf spent close to the dam did not differ between partially covered pens and uncovered pens (1,047 ± 24.1 vs. 1,089 ± 28.7 min/24 h, respectively; χ^2^_1_ = 1.23; *P* = 0.268).

This study aimed to determine whether dairy calves housed in individual pens with their dam used an area of cover to hide during the first 3 d after birth, and to determine whether the provision of cover affected maternal and neonatal behavior. The calves did not show a preference for the covered side of the pen and thus did not show hiding behavior. However, calves housed in covered pens received more maternal sniffing and licking than calves in uncovered pens. Overall, calves spent less time lying down on d 1 than d 2 and 3. As days increased after birth, calves received less sniffing and licking, and cows spent less time standing with head over calf and in close proximity of calf.

We hypothesized that calves housed in the partially covered pen would spend more time in the covered side of the pen, but detected no such preference. Proudfoot et al. (2014) found that 79% of the cows housed in a partially covered pen calved on the covered side, suggesting that cows seek seclusion at calving. Other studies suggest that calves housed in group pens are motivated to seek seclusion during the first hours after birth (Jensen and Rørvang, 2018; Zobel et al., 2020). In the present study, all cow-calf pairs were already separated from the herd and housed in individual calving pens, which may explain lack of hiding behavior of the calves. Furthermore, in the present study we defined being positioned in that half of the pen that was covered toward the group pen as being secluded, but since the “open” side of the partially covered pen was covered toward the neighboring pen, calves may have perceived both sides as equally secluded. The distance of the calf to the pen side, when lying, was unfortunately not recorded, but causal observation suggests that most calves were positioned close to a pen wall when lying.

Our second hypothesis was that calves housed in a partially covered pen receive more maternal care than calves housed in the uncovered pen. In accordance with this hypothesis, calves in the partially covered pens received more sniffing and licking than calves housed in an uncovered pen. One explanation for this may be that if the cow perceives the calf as adequately hidden (from herd mates and potential predators) by the provided cover, she is calmer and more motivated to perform maternal behavior. Another explanation may be that the cow has a preference for staying in the covered side (Proudfoot et al., 2014) and that accommodating a secluded area reduces cows' vigilance and therefore increases maternal behavior. Sniffing and licking supports bonding between cow and calf (Kendrick et al., 1997) and benefits the newborn calf by stimulating breathing, circulation, urination, and defecation (Metz and Metz, 1986). Previous studies reported that sniffing and licking are most intense during the first 6 h after birth and decline steadly thereafter (e.g., Edwards and Broom, 1982; Jensen, 2011). In agreement, the duration of sniffing and licking was significantly higher during the first day after birth compared with later days in the current study.

We also expected calves in covered pens to spend more time suckling, but found no support for this expectation. The duration of suckling recorded by instantaneous sampling in this study is within the same range as the duration of suckling observed by Jensen (2011) using continuous recording. The assisted suckling events that occurred on d 1 were excluded due to human disturbance and therefore the duration of suckling is likely underestimated on d 1. However, as a similar proportion of calves on the 2 treatments were assisted, this is unlikely to affect the results.

There was no difference in duration of time calves spent lying between pen types, and lying time is within the same range as found by Jensen (2011). Calves were lying least on the first day, which is likely due to being active during the first hours after birth using several attempts to stand and searching for the udder (Edwards and Broom, 1982; Campler et al., 2015). On the other hand, the higher lying time on d 2 and 3 may be linked to cattle being a hider species and calves spending most their time lying hidden during the first days after birth. According to Jensen (2011), the duration of lying in calves decreased from d 3 to 11 after birth, suggesting that calves become increasingly more active starting at d 3 after birth, possibly coinciding with joining the herd (Vitale et al., 1986).

The third hyphothesis was that cows housed in a partially covered pen allow a longer distance to their calf and show less protective behavior, but we found no support for this hyphothesis. It should be noted that there were high individual differences in protective behavior between cows. Individuals within a herd show different levels of maternal aggressiveness, which has been found to be consistent over time (Haskell et al., 2014) and there is most likely an effect of personality on maternal behavior. Breeding dairy cows for temperament traits that favor the expression of wanted maternal behavior, such as high maternal care, but low maternal defensiveness to reduce the potential risk of injury to stockpeople, may be warranted.

In addition to the duration of protective behavior, the cow-calf distance is an indicator of the cow's feeling of security. It is mainly the cow who maintains the proximity to her calf (Edwards and Broom, 1982) and the cow approaches the calf when she perceives it to be in imminent danger (Vitale et al., 1986). No effect of pen type could be detected; thus, we found no support for the hypothesis that partially covered calving pens increased cows' sense of security. The cow-calf distance increased over study days, which is in accordance with Edwards and Broom (1982) and supports the classification of cattle as a hider species, since follower species would be assumed to stay close throughout this early period of life.

In conclusion, the provision of cover in an individual calving pen increased maternal sniffing and licking. This result provides some support that providing cover in the few days after birth enhances maternal behavior and may improve bonding between the cow and her calf. Sniffing and licking, as well as protective behavior and proximity, were highest during the first day after birth and decreased thereafter.
